# SOX2‐positive retinal stem cells are identified in adult human pars plicata by single‐cell transcriptomic analyses

**DOI:** 10.1002/mco2.198

**Published:** 2022-12-24

**Authors:** Xiaotang Wang, Wei Fan, Zongren Xu, Qi Zhang, Na Li, Ruonan Li, Guoqing Wang, Siyuan He, Wanqian Li, Dan Liao, Zhi Zhang, Nan Shu, Jiaxing Huang, Chenyang Zhao, Shengping Hou

**Affiliations:** ^1^ The First Affiliated Hospital of Chongqing Medical University Chongqing China; ^2^ Chongqing Key Laboratory of Ophthalmology Chongqing China; ^3^ Chongqing Eye Institute Chongqing China; ^4^ Chongqing Branch (Municipality Division) of National Clinical Research Center for Ocular Diseases Chongqing China; ^5^ Department of Biochemistry and Molecular Biology College of Basic Medicine Chongqing Medical University Chongqing China

**Keywords:** retinal degeneration, retinal stem cell, scRNA‐seq, SOX2^+^AQP1^+^TSPAN12^+^

## Abstract

Stem cell therapy is a promising strategy to rescue visual impairment caused by retinal degeneration. Previous studies have proposed controversial theories about whether in situ retinal stem cells (RSCs) are present in adult human eye tissue. Single‐cell RNA sequencing (scRNA‐seq) has emerged as one of the most powerful tools to reveal the heterogeneity of tissue cells. By using scRNA‐seq, we explored the cell heterogeneity of different subregions of adult human eyes, including pars plicata, pars plana, retinal pigment epithelium (RPE), iris, and neural retina (NR). We identified one subpopulation expressing SRY‐box transcription factor 2 (SOX2) as RSCs, which were present in the pars plicata of the adult human eye. Further analysis showed the identified subpopulation of RSCs expressed specific markers aquaporin 1 (AQP1) and tetraspanin 12 (TSPAN12). We, therefore, isolated this subpopulation using these two markers by flow sorting and found that the isolated RSCs could proliferate and differentiate into some retinal cell types, including photoreceptors, neurons, RPE cells, microglia, astrocytes, horizontal cells, bipolar cells, and ganglion cells; whereas, AQP1^−^ TSPAN12^−^ cells did not have this differentiation potential. In conclusion, our results showed that SOX2‐positive RSCs are present in the pars plicata and may be valuable for treating human retinal diseases due to their proliferation and differentiation potential.

## Graphical abstract

SOX2‐positive retinal stem cells (RSCs) are identified in adult human pars plicata. Using the specific surface markers aquaporin 1 and tetraspanin 12, the RSCs were isolated and proved to have proliferation and differentiation abilities.

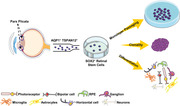

## 1 INTRODUCTION

Retinal degeneration is a major cause of vision loss and blindness worldwide, including age‐related macular degeneration, retinitis pigmentosa, Stargardt's disease, glaucoma, and ischemic optic neuropathy.^1^, ^2^ In these diseases, pathological damage may lead to the eventual death of retinal cells such as retinal pigment epithelial (RPE) cells, photoreceptors, and retinal ganglion cells. Stem cells refer to cells with the capacity for long‐term self‐renewal and the ability to differentiate into various cell types (multipotent). Adult stem cells have been broadly recognized to be present in a variety of human organs and tissues.^3^ ^—^ ^6^ Stem cell therapy would be an effective way to restore visual function, but whether in situ retinal stem cells (RSCs) that can differentiate into other retinal cells exist in the mammalian retina is currently controversial.

The retina develops from the optic cup formed by the neuroectoderm, which is an extension of the brain and part of the central nervous system (CNS). As the most common neural stem cell marker, SRY‐box transcription factor 2 (SOX2) is reported to be important for the development of the visual system.^7^, ^8^ In 2000, Tropepe et al.^9^ first proposed that RSCs exist in the mammalian (mouse) pigmented ciliary margin. The ciliary body contains the pars plicata and pars plana and is derived from the optic cup (neurectoderm), including the outer pigmented layer and the inner non‐pigmented layer. The outer pigmented layer is connected to the RPE, and the inner non‐pigmented layer is connected to the neural retina (NR). In 2004, Coles et al.^10^ further proved the existence of adult RSCs in the human ciliary crown; however, in 2009, Cicero et al.^11^ proposed the opposite claim and reported that the cells previously proven to be RSCs were actually pigmented ciliary epithelial cells. Whether RSCs exist in human eye tissue remains controversial, and the specific markers of RSCs are still unknown.

Single‐cell RNA sequencing (scRNA‐seq) technologies offer high‐throughput sequencing analysis of the genome, transcriptome, and epigenome at the single‐cell level. Cutting‐edge high‐throughput scRNA‐seq technology provides opportunities for revolutionizing our understanding of heterogeneous cell subtypes, discovering new cell types, and identifying unique cell states at an unprecedented resolution.^12^ ^—^ ^16^ Our previous scRNA‐seq study of human RPE demonstrated the heterogeneity and molecular map of hRPE cells.^17^ We also demonstrated the important role of retinal microglia in the development of experimental autoimmune uveitis by scRNA‐seq.^18^ Current knowledge of human RSCs is mostly based on broad investigations at the whole tissue and bulk cell population levels.^19^ ^–^ ^21^ Single‐cell transcriptomics holds promise for uncovering novel types of RSCs from heterogeneous eye tissues.

In the present study, we performed scRNA‐seq using cells isolated from five tissues of the human eyeball to identify the types/subtypes of cells and to define RSCs at single‐cell resolution. We identified an SOX2^+^ RSC population in the pars plicata of the ciliary body. The genes aquaporin 1 (AQP1) and tetraspanin 12 (TSPAN12) were selected as specific surface markers for SOX2‐positive RSCs and used to sort out the identified RSCs by flow cytometry. We further validated the proliferation and differentiation abilities of these SOX2‐positive RSCs, providing new evidence for the existence of RSCs in adult human eyes.

## 2 RESULTS

### 2.1 Identification of the SOX2‐positive human RSC subpopulation

**FIGURE 1:**
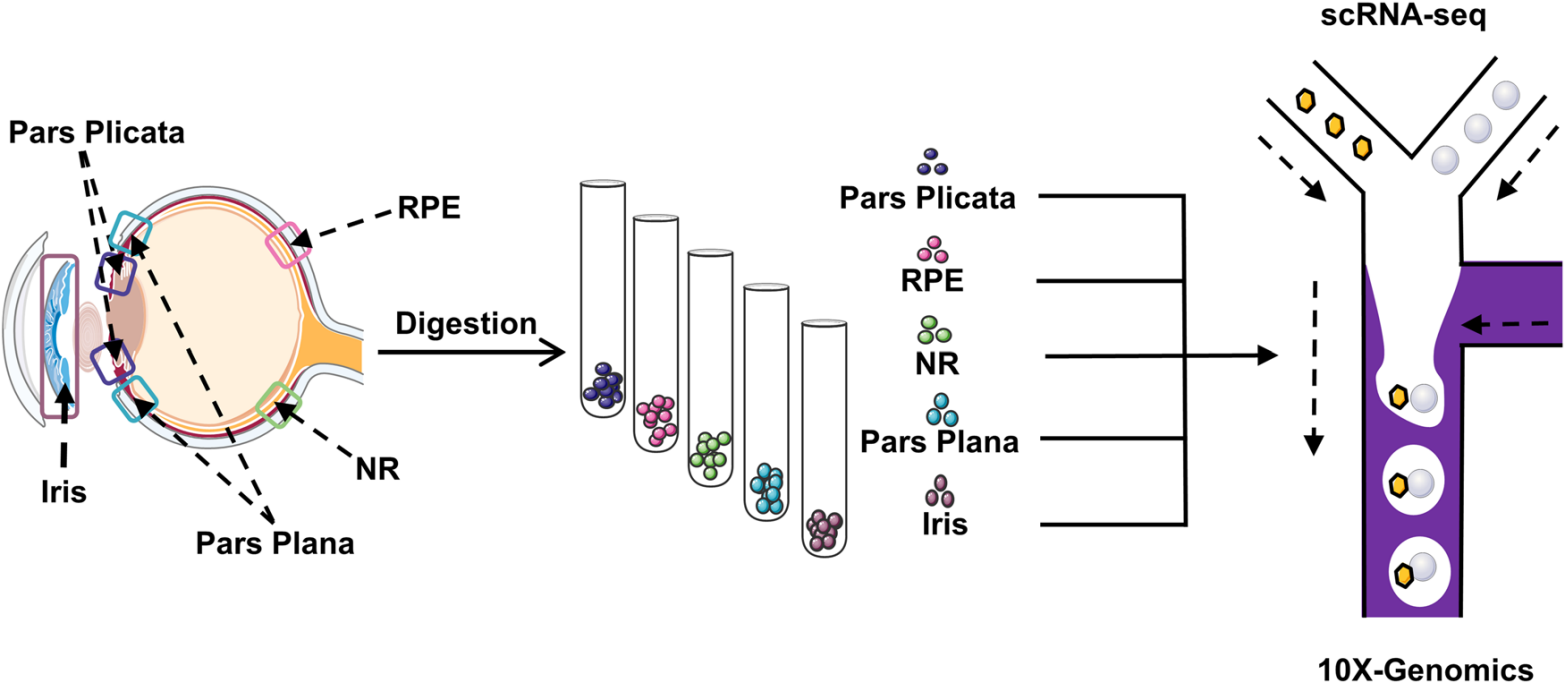
Workflow of the isolation of cells from five areas of healthy human eye tissues for single‐cell RNA (scRNA)‐sequation (drawn with Adobe Illustrator)

**FIGURE 2:**
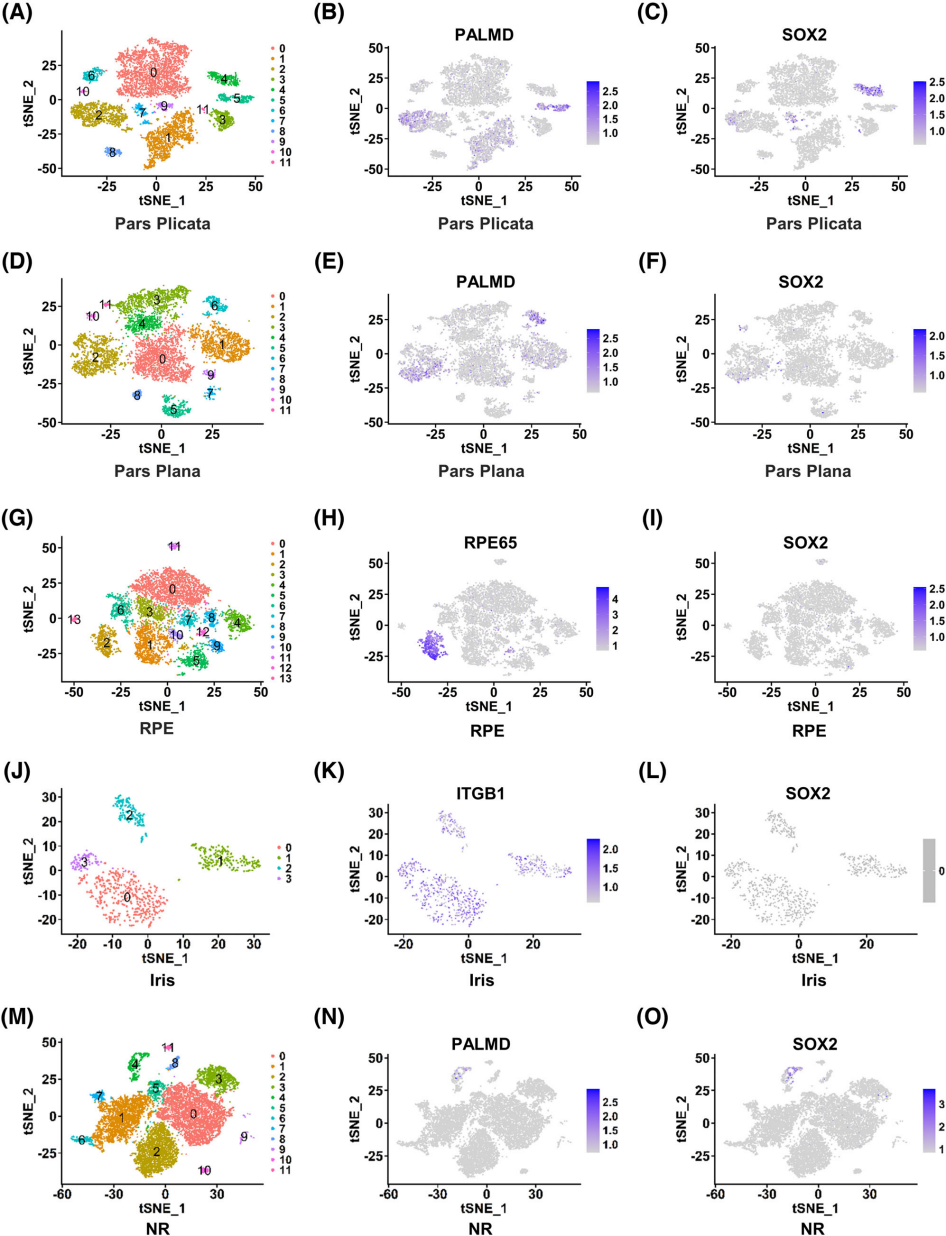
Single‐cell profiling revealed cell heterogeneity in five tissues (the pars plicata, pars plana, retinal pigment epithelium [RPE], iris, and neural retina [NR]). (A) *t*‐Distributed stochastic neighbor embedding (*t*‐SNE) plot shows the heterogeneity of the pars plicata. (B) Expression distribution of PALMD in t‐SNE plots for pars plicata samples. (C) Expression distribution of SOX2 in t‐SNE plots for pars plicata samples. (D) t‐SNE plot shows the heterogeneity of the pars plana. (E) Expression distribution of PALMD in t‐SNE plots for pars plana samples. (F) Expression distribution of SOX2 in t‐SNE plots for pars plana samples. (G) The t‐SNE plot shows the heterogeneity of the RPE. (H) Expression distribution of RPE65 in t‐SNE plots for RPE samples. (I) Expression distribution of SOX2 in t‐SNE plots for RPE samples. (J) t‐SNE plot shows the heterogeneity of the iris. (K) Expression distribution of ITGB1 in t‐SNE plots for iris samples. (L) Expression distribution of SOX2 in t‐SNE plots for iris samples. (M) t‐SNE plot shows the heterogeneity of the NR. (N) Expression distribution of PALMD in t‐SNE plots for NR samples. (O) Expression distribution of SOX2 in t‐SNE plots for NR samples

To explore whether in situ RSCs are present in the adult human eye, scRNA‐seq technology was used to perform transcriptome analysis of five areas of the adult human eye including pars plicata, pars plana, RPE, iris, and NR (Figure 1). The pars plicata sample with 7671 cells could be divided into 12 clusters (Figure 2A). Interestingly, cluster 4 in the pars plicata, which included 384 cells, specifically expressed the neural stem cell marker gene SOX2, and other clusters expressed the differentiated ciliary epithelium marker gene PALMD (Figure 2B,C). The pars plana sample with 7392 cells included 12 clusters, and all clusters expressed the differentiated ciliary epithelium marker gene PALMD (Figure 2D,E). However, there was no expression of SOX2 in the pars plana (Figure 2F). The tSNE plots of the RPE sample (7876 cells) and iris sample (841 cells) were divided into 14 clusters and four clusters, respectively (Figure 2G,J). The RPE marker gene RPE65 was specifically expressed in cluster 2 in the RPE sample, and the iris marker gene ITGB1 was specifically expressed in all clusters in the iris sample (Figure 2H,K). However, there was no expression of SOX2 in either sample (Figure 2I,L). The NR sample with 12,742 cells consisted of 12 clusters (Figure 2M). SOX2 and PALMD were both slightly expressed in cluster 4 (Figure 2N,O). We hypothesized that the NR tissue was mixed with a small number of pars plicata cells because of their adjacent anatomical relationship. In conclusion, we found that SOX2 was specifically expressed in cluster 4 in the pars plicata sample, which may represent the subpopulation of RSCs.

### 2.2 Functional analyses of the identified RSC cluster

**FIGURE 3:**
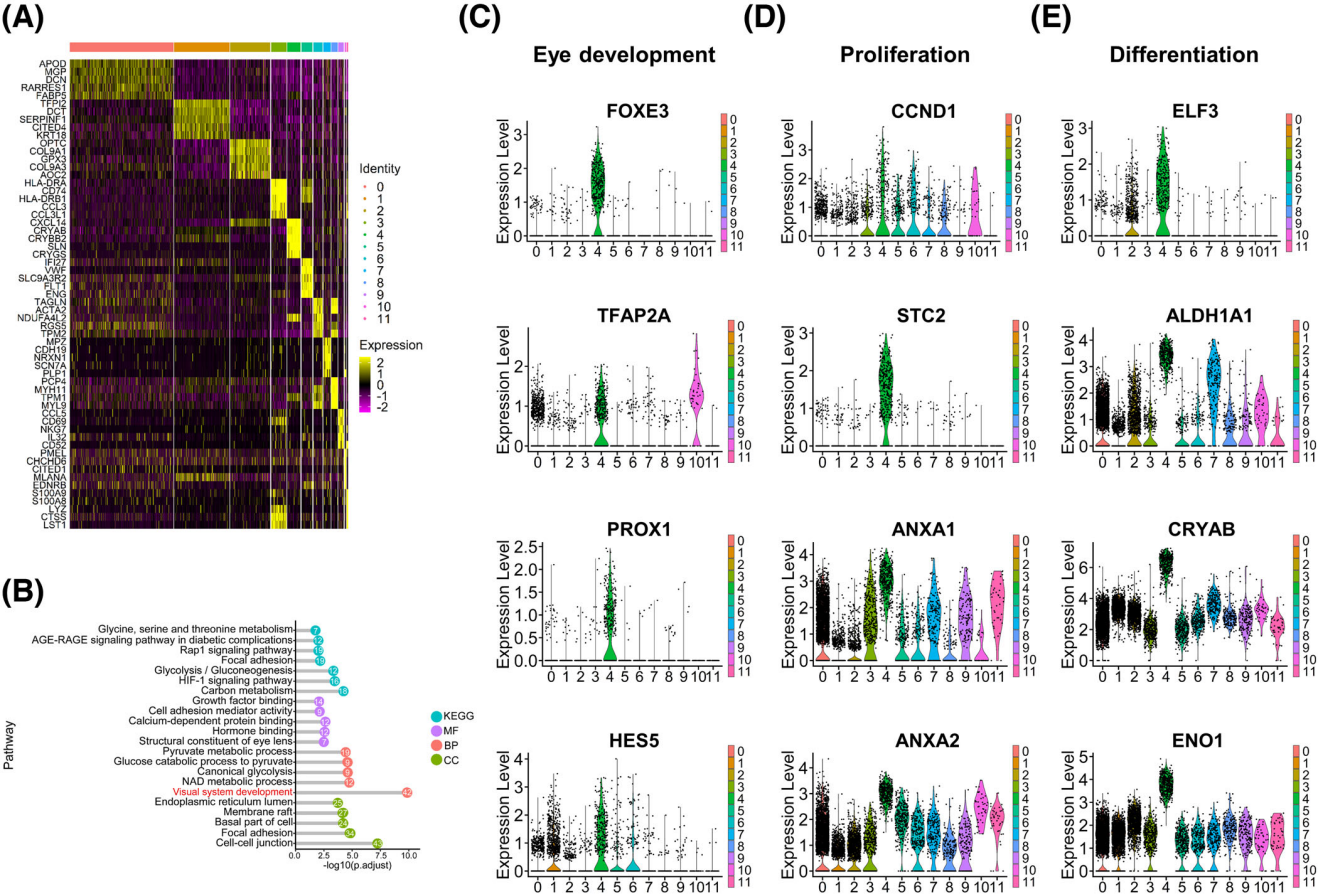
Functional analyses of the identified retinal stem cell (RSC) cluster. (A) Heatmap showing the differences in gene expression among 12 clusters in pars plicata. (B) KEGG and GO pathway enrichment analyses of differentially expressed genes of cluster 4 in pars plicata. (C–E) Violin plots showing marker gene expression related to eye development, proliferation, and differentiation

To further establish the molecular differences among the 12 clusters of the pars plicata, we compared the gene expression in each cluster against all other clusters in the pars plicata. The top 5 differentially expressed gene (DEG) markers that distinguished each cluster from the others are shown in the heatmap (Figure 3A). Dynamic metabolic patterns are important characteristics of stem cells.^22^, ^23^, ^24^, ^25^ We found that most pathways of cluster 4 were related to the regulation of metabolism by KEGG analyses, suggesting the important characteristics of stem cells for cluster 4 (Figure 3B). GO analyses also indicated that the DEGs of the identified RSC cluster 4 were related to visual system development processes (Figure 3B). To further identify the functional roles of cluster 4, we examined the expression of an expanded set of genes related to cell properties in eye development (FOXE3,^26^ TFAP2A,^27^ PROX1,^28^ and HES5^29^), proliferation(CCND1,^30^, ^31^ STC2,^32^ ANXA1,^33^ and ANXA2^34^) and differentiation (ELF3,^35^ ALDH1A1,^36^ CRYAB,^37^ and ENO1^38^) (Figure 3C–E), and the results showed that these aforementioned genes were highly expressed in cluster 4 and further demonstrated the stem cell features of the identified RSC cluster.

### 2.3 AQP1 and TSPAN12 double‐positive cells may represent RSCs

**FIGURE 4:**
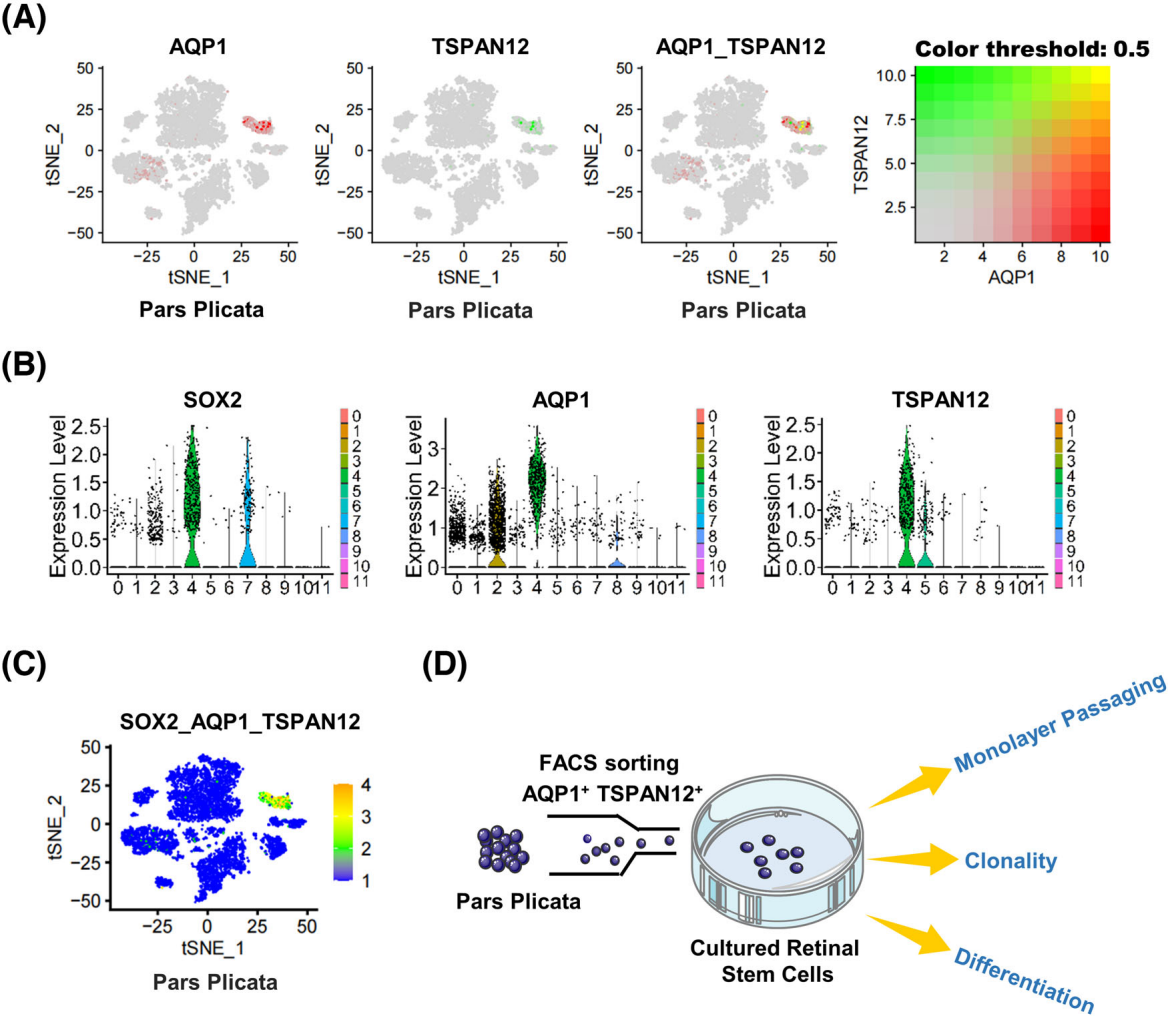
Fluorescence‐activated cell sorting (FACS) sorting of stem cells by specific and differentially expressed marker genes in plicata samples. (A) Expression patterns of aquaporin 1 (AQP1) and tetraspanin 12 (TSPAN12). Red represents high expression levels of AQP1, green represents high expression levels of TSPAN12, and yellow represents mixed high expression levels. (B) Violin plot shows AQP1, TSPAN12, and SOX2 marker genes for pars plicata samples. (C) Average expression of AQP1, TSPAN12, and SOX2 was projected on the t‐distributed stochastic neighbor embedding (*t*‐SNE) plot to identify the stem cell population. Orange indicates maximum gene expression, while blue indicates low or no expression of a particular set of genes in log‐normalized UMI counts. (D) Workflow of stemness verification after FACS sorting (drawn with Adobe Illustrator)

The sequencing data showed that the cluster 4 population contained 384 cells, of which SOX2‐positive cells accounted for 76.04% (3.80% of total pars plicata cells). As a transcription factor, SOX2 localizes to the nucleus and has been shown to be necessary for the long‐term self‐renewal of neural stem cells.^39^ For further stemness validation of SOX2‐positive RSCs without destroying their cell structure, we screened SOX2‐positive RSCs in cluster 4 by the specific surface markers AQP1 and TSPAN12 according to scRNA‐seq data (Figure 4A–C). AQP1 and TSPAN12 were highly and especially expressed in cluster 4. AQP1 was previously reported to be upregulated in undifferentiated human embryonic stem cells (hESCs) and human induced pluripotent stem cells (hiPSCs), as well as hESC‐ and hiPSC‐derived RPE cells.^40^ TSPAN12 could accelerate mitotic progression to regulate proliferation, one of the stemness characteristics, by controlling the cell cycle.^41^ The specific surface markers AQP1 and TSPAN12 accounted for 70.31% of cluster 4 (3.52% of pars plicata cells), which is consistent with the percentage of the SOX2‐positive subpopulation. Then, SOX2‐positive RSCs were isolated by flow cytometry using the specific surface marker genes AQP1 and TSPAN12. The isolated cells accounted for approximately 1% of the whole pars plicata (Figure S1). The number of isolated AQP1 and TSPAN12 double‐positive cells was in a reasonable range according to the sequencing results. The flow chart of the stemness verification experiments of double‐positive cells after flow sorting is shown in Figure 4D.

### 2.4 SOX2‐positive cells in the pars plicata have proliferation potential

**FIGURE 5:**
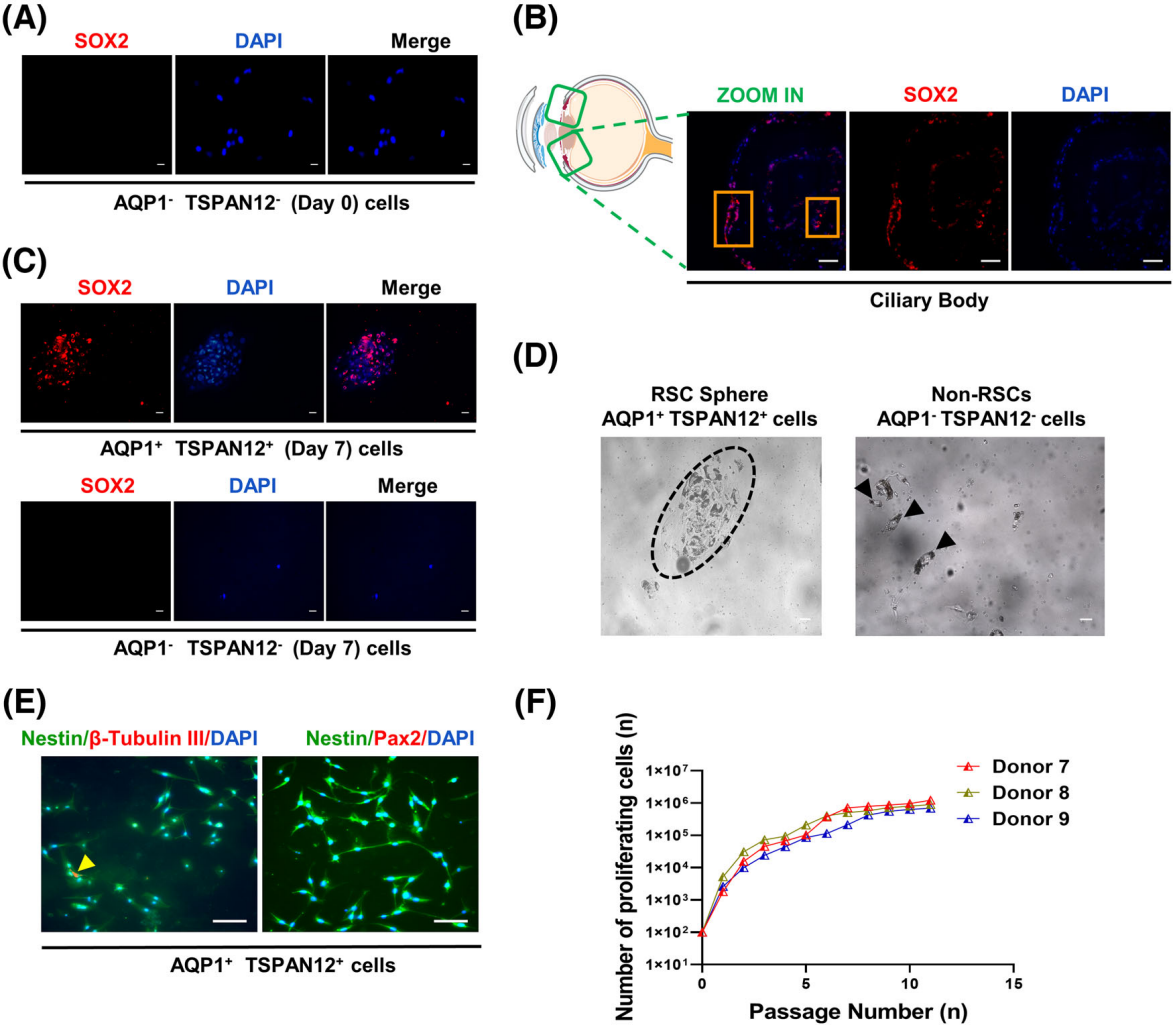
Verification of the proliferation potential of SOX2‐positive cells in the pars plicata. (A) SOX2 and 4′,6‐diamidino‐2‐phenylindole (DAPI) immunofluorescence of aquaporin 1 (AQP1) and tetraspanin 12 (TSPAN12) double‐negative cells isolated from the pars plicata. (B) In situ immunostaining of SOX2 in pars plicata sections. Orange boxes denote the SOX2‐positive cells in the pars plicata. The schematic image was drawn by Adobe Illustrator. (C) SOX2 and DAPI immunofluorescence of AQP1 and TSPAN12 double negative/positive cells in the pars plicata after 7 days of suspension culture. (D) The observation of AQP1 and TSPAN12 double negative/positive cells after 7 days of suspension culture. The black dotted line box denotes double‐positive cells suspended into a sphere. The black arrows denote double‐negative cells that cannot be suspended into a sphere. (E) Colocalization of Nestin/β‐Tubulin III and Nestin/Pax2 by double‐label immunofluorescence. (F) Proliferation abilities of the isolated SOX2‐positive cells in the pars plicata are capable of long‐term self‐renewal, as demonstrated by monolayer passaging of sorted AQP1 and TSPAN12 double‐positive cells. Each line represents the expansion of SOX2‐positive retinal stem cells (RSCs) from three different donor eyes. Scale bars: 10 μm (A, C, and D), scale bars: 50 μm (B, E)

Since proliferation is one of the characteristics of stemness, we tested the proliferation ability of isolated AQP1 and TSPAN12 double‐positive cells. First, we sorted AQP1 and TSPAN12 double‐negative cells in the pars plicata and performed SOX2 immunofluorescence staining as a negative control. We found that AQP1 and TSPAN12 double‐negative cells did not express the neural stem cell marker SOX2 (Figure 5A). At the same time, we performed SOX2 in situ immunofluorescence staining on the eyeballs of the donors, and the experimental results showed that there were indeed scattered SOX2‐positive cells in the pars plicata (Figure 5B). Then we conducted a suspension culture of the isolated AQP1 and TSPAN12 double‐positive cells and double‐negative cells for 7 days and found that the double‐positive cells were positive for SOX2 and could be suspended into spheres, while the double‐negative cells could not (Figure 5C,D), suggesting that AQP1^+^TSPAN12^+^ cells had proliferation potential. Asymmetric division is another characteristic of adult stem cells. We performed SOX2 immunofluorescence staining on the suspension spheres and determined the SOX2‐positive rate. The results showed that the SOX2‐positive rate in the suspension spheres ranged from 37% to 45% (Table 1), which is consistent with the characteristic asymmetric division of adult stem cells. Then, we carried out immunofluorescence staining of Nestin (a retinal progenitor marker), β‐Tubulin III (an early neuron marker), and Pax2 (a mature glial marker) in suspension spheres. We observed that the isolated AQP1^+^TSPAN12^+^ cells were positive for Nestin and β‐Tubulin III but negative for Pax2 (Figure 5E). These results showed that double‐positive cells had a tendency to gradually differentiate into neurons from progenitor cells rather than incorporate them into the isolated retinas. We carried out a monolayer subculture of the identified RSCs after flow sorting. The results showed that the double‐positive cells could be subcultured and proliferate, as shown in Figure 5F, suggesting that the double‐positive cells had proliferation potential.

**TABLE 1:** The percentage of SOX2‐positive cells after 7 days of suspension culture of aquaporin 1 (AQP1) and tetraspanin 12 (TSPAN12) double‐positive cells from six different donor eyes

**Donors**	**Age (years)**	**Gender**	**Spheres (cells/μl)**	**SOX2^+^ (%)**
Donor 7	40	Male	81	43.75
Donor 8	42	Male	74	42.11
Donor 9	45	Female	103	41.62
Donor 10	48	Male	98	37.13
Donor 11	50	Female	87	45.74
Donor 12	53	Female	69	39.30

### 2.5 SOX2‐positive pars plicata cells have differentiation potential

**FIGURE 6:**
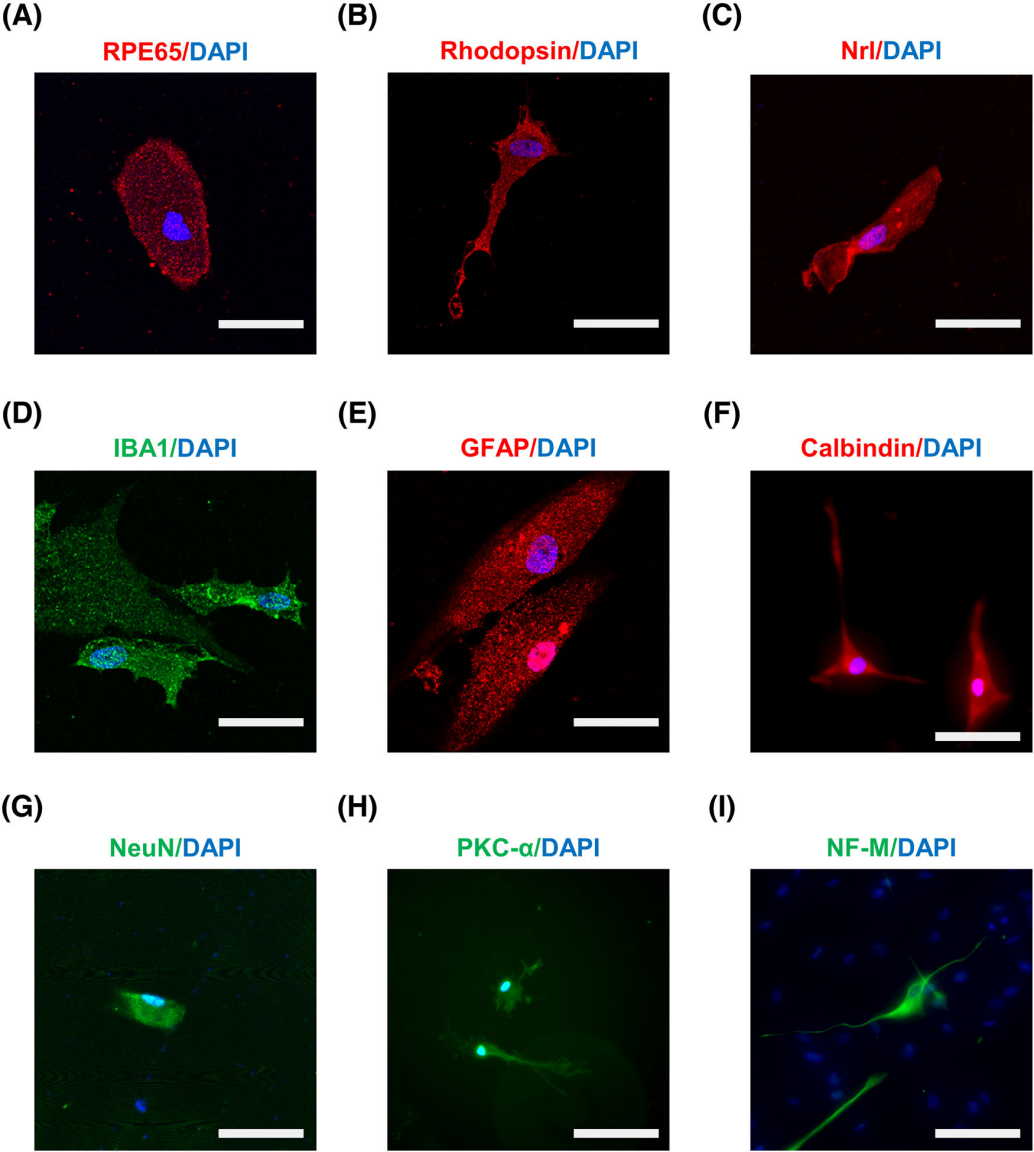
Multipotentiality verification. After culturing in differentiation media for 3 weeks, the differentiation abilities of the isolated cells were assayed by immunofluorescence staining of different cell markers. The nuclei of all cells were stained with 4′,6‐diamidino‐2‐phenylindole (DAPI). (A) Retinal pigment epithelium (RPE) cells (RPE65^+^). (B‐C) Photoreceptor cells (Rho^+^, Nrl^+^). (D) Microglia (IBA1^+^). (E) Astrocytes (GFAP^+^). (F) Horizontal cells (Calbindin^+^). (G) Neurons (NeuN^+^). (H) Bipolar cells (PKC‐α^+^). (I) Ganglion cells (NF‐M^+^). Scale bars: 10 μm (A–E); 25 μm (F–I)

To further confirm the stemness of SOX2‐positive RSCs from pars plicata of human eyes, we tested the cell differentiation potential. SOX2‐positive RSCs from pars plicata were cultured in the same differentiation media for the differentiation experiments according to previous studies,^9^, ^10^, ^42^, ^43^, ^44^ and the differentiated cells were stained with immunofluorescence for different cell types of the RPE and the NR. After culturing for 3 weeks, we observed that the isolated cells were positive for markers of various retinal cells with different morphologies, including RPE65 (a marker of the RPE, Figure 6A), Nrl/Rho (markers of photoreceptors, Figure 6B,C), IBA1 (a marker of microglia, Figure 6D), GFAP (a marker of astrocytes, Figure 6E), calbindin (a marker of horizontal cells, Figure 6F), NeuN (a marker of neurons, Figure 6G), PKC‐α (a marker of bipolar cells, Figure 6H) and NF‐M (a marker of ganglion cells, Figure 6I). The differentiation ratio of SOX2‐positive RSCs after being cultured in differentiation media for three weeks was determined, and the results showed that the majority of the differentiated cells were photoreceptor cells (as indicated by the presence of Nrl and rhodopsin staining) and a minority of the differentiated cells were RPE cells (as indicated by the presence of RPE65 staining) (Table 2 and Figure S2). These results indicate the differentiation abilities of the isolated SOX2‐positive RSCs.

**TABLE 2:** The percentage of cell types after differentiation culture

**Cell type**	**Markers**	**Percent positive** **(%)**	**Total cell numbers** **(DAPI^+^)**
RPE	RPE65	2.0 ± 1.3	617
Photoreceptor	Rhodopsin/Nrl	24.7 ± 8.5	436
Microglia	IBA1	3.7 ± 2.1	342
Astrocytes	GFAP	12.6 ± 7.0	264
Horizontal cells	Calbindin	9.1 ± 5.5	389
Neurons	NeuN	10.6 ± 4.2	268
Bipolar cells	PKC‐α	5.9 ± 3.2	201
Ganglion cells	NF‐M	2.6 ± 1.7	464

## 3 DISCUSSION

Details of the cell types and gene expression patterns of many complex tissues can be better elucidated by single‐cell sequencing technology. Previous studies on RSCs have been performed only at the tissue level, and the existence of RSCs is still controversial.^10^, ^11^ Therefore, we intended to refine the scope of studies on RSCs and tried to determine whether RSCs exist at the cellular level by scRNA‐seq of five areas in adult human eyes.^45^, ^46^, ^47^, ^48^, ^49^ Our results showed that pars plicata tissues could be categorized into 12 clusters, one of which highly expressed the neural stem cell marker SOX2 with low expression of the differentiated ciliary epithelium marker PALMD, which was consistent with the characteristics of adult stem cells with high expression of stem cell markers and low expression of adult tissue cell markers. In addition, the identified SOX2‐positive RSCs possessed the ability to proliferate and differentiate. Interestingly, SOX2‐positive RSCs differentiated into photoreceptor cells with the highest proportion and differentiated into RPE with the lowest proportion, which is consistent with previous studies.^10^ These findings strongly indicate that RSCs are present in the human pars plicata, as reported by Coles et al.^10^

The transcription factor SOX2 plays a critical role in the development of the visual system and brain.^50^, ^51^ Patients who carry mutations in SOX2 display severe visual impairment.^52^ In human eyes, SOX2 acts in neuronal progenitors to regulate glial differentiation fate.^51^ Moreover, the self‐renewal of neural stem cells depends on SOX2.^39^ Since the retina is a CNS tissue, we suppose that the functions of the retina will also be regulated by SOX2. Intriguingly, SOX2 has been reported to participate in the regulation of neuroectoderm differentiation,^53^ which further supports our hypothesis about the stemness potential of SOX2‐positive RSCs in human eyes.

AQPs are a family of water‐transporting membrane channel proteins. AQP1 is one of the AQPs and has been reported to play roles in multiple biological processes related to cell proliferation,^54^ migration,^55^ and inflammation.^56^ Recent studies have also indicated the role of AQP1 in regulating stem cells. Decreased AQP1 expression was found to be related to aged tendon stem/progenitor cells (TSPCs) by activating JAK‐STAT signaling pathways and overexpression of AQP1 restored the self‐renewal of TSPCs.^57^ Mesenchymal stem cell function of migration is largely dependent on AQP1 and C‐X‐C chemokine receptor type 4, which activates the Akt and Erk intracellular signaling pathways.^58^ Moreover, there exists a correlation between the aquaporin expression profile and neural differentiation.^59^ Additionally, TSPAN12 has been reported to be a proliferation‐promoting regulatory factor in a variety of neoplastic diseases, including small‐cell lung carcinoma, non‐small cell lung carcinoma, ovarian cancer, and hepatocellular carcinoma.^41^, ^60^, ^61^, ^62^ Furthermore, TSPAN12 functions as an important regulator of retinal vascular development by promoting Norrin‐induced FZD4/beta‐catenin signaling.^63^ Previous studies combined with our results suggest that AQP1 and TSPAN12 are expected to be potential proliferation markers for SOX2‐positive RSCs. Further knockout studies for AQP1 and TSPAN12 are needed to elucidate the key role of these two genes in the regulation of cell fate.

As endogenous stem cells, RSCs represent the self‐regenerative capacities of the tissue throughout its lifespan. Identifying endogenous stem cells is a challenging but important task. The local environment of endogenous stem cells, referred to as the niche, is essential for maintaining the quantity and quality of stem cells available for renewal and regeneration. Understanding the niche of stem cells is essential for reducing adverse outcomes of degenerative diseases. Compared with transplantation, endogenous stem cells overcome the disadvantages of poor accessibility, transplant rejection, and the use of immunosuppressants.^64^ Currently, therapies for retinal degeneration diseases are quite limited, indicating the urgent need to explore new therapies. We identified a subpopulation of SOX2‐positive RSCs in the pars plicata that could differentiate into other retinal cells. Based on the flow cytometry assay, the sorted SOX2‐positive RSCs with the specific markers AQP1 and TSPAN12 may help to cure retinal degeneration diseases by regenerating retinal cells. Our results indicate that the pars plicata exhibits an important physiological function as an ecological niche for SOX2‐positive RSCs, which provides new insight into treating blinding eye diseases with endogenous RSCs.

However, there are still a few limitations in our study. First, although we proved that SOX2‐positive RSCs are present in the pars plicata, how to stimulate these cells to proliferate and differentiate into functional cells in the retina in situ remains unknown. Second, although experiments validating the stemness of SOX2‐positive RSCs in vitro were conducted, whether these cells retain their stemness properties when they are transplanted into the retina needs further investigation.

In summary, we identified a subpopulation of SOX2‐positive cells in the pars plicata as adult RSCs and examined the proliferation and differentiation abilities of these cells, which may provide new therapeutic strategies for retinal degeneration diseases.

## 4 MATERIALS AND METHODS

### 4.1 Samples and preparation of single‐cell suspensions

Eyeballs from six donors were divided into five tissue regions within 32 h including the iris, pars plicata, pars plana, RPE, and neuroepithelial layer (Donors 1–6, Table S1). The samples were finely minced with scalpels and dissociated in a buffer containing papain (20 U/ml, LS003119; Worthington) supplemented with DNase‐I (2000 U/ml, 10104159001; Roche) in Earle's Balanced Salt Solution at 37°C for 30 min. The cell suspensions were resuspended in Earle's Balanced Salt Solution and passed through a 30‐μm cell strainer. Then, the viability of the samples was evaluated with AO/PI prior to loading on the single‐cell platform. The samples displayed 90% viability on average.

### 4.2 Single‐cell sequencing

Single‐cell cDNA libraries of sample pools were generated using the 10X Genomics platform following the manufacturer's instructions (Document Number CG000206). Briefly, single‐cell suspensions were loaded into 10X Chromium according to the kit instructions, followed by cDNA amplification and library construction according to standard procedures. Qubit was used for library quantification before pooling. The final library pool was sequenced on an Illumina Novaseq6000 instrument using 150 base‐pair paired‐end reads.

### 4.3 scRNA‐seq data analysis

The Cellranger (version 3.1.0) pipeline with the default and recommended parameters was used for preprocessing and sequence alignment of data reads.^65^ The human reference genome used for the alignment was GRCh38. Next, Gene‐Barcode matrices were generated for each individual sample by counting unique molecular identifiers (UMIs) and filtering noncell‐associated barcodes. Finally, the gene‐barcode matrix was generated by the pipeline, and it contained the barcoded cells and gene expression counts.

After Cellranger processing, the count matrix, barcode information, and gene information of each sample were imported into R software (version 3.6.1), and each sample was analyzed separately according to the standard process of Seurat (version 3.1.5).^66^ First, we performed preliminary filtering of the imported data, and the filtered data (features detected in at least three cells, cells in which at least 200 features were detected) were used for subsequent analysis. Next, to avoid the existence of low‐quality cells interfering with subsequent results, we removed outlier cells. Doublet‐cell prediction was performed on these cells by the R package DoubletFinder (version 2.0.3),^67^ and doublet cells that could affect the clustering were removed. Therefore, we obtained 7671 cells in the plicata sample, 7392 cells in the plana, 7876 cells in the RPE, 12742 cells in the NR, and 841 cells in the iris. Second, the cell cycle of each sample was analyzed, and it did not affect the grouping of the samples. After the above analyses, we normalized the data by setting the parameter scale factor = 10,000, and the normalization method was LogNormalize. We also obtained the highly variable features by setting the parameter nfeatures = 2000 with the method “vst”. Similarly, we scaled and centered features in the dataset with the function ‘ScaleData’, and we regressed out confounding factors: number of UMIs, number of features, percentage of mitochondrial RNA, and percentage of hemoglobin RNA. Third, we performed a principal component analysis of the processed dataset to achieve dimensionality reduction, and the selection of these PCs was based on elbow and jackstraw plots. Clusters were determined by a clustering algorithm based on shared nearest neighbor (SNN). Additionally, the resolution parameter for clustering was 0.3. Finally, we chose *t*‐distributed stochastic neighbor embedding (*t*‐SNE) to visualize our clustering results. To obtain the differentially expressed genes of each cluster, we conducted a differential analysis for each cluster with the function ‘Find Markers’ and the parameter ‘Wilcox’.

### 4.4 Pars plicata section immunostaining

First, all pars plicata were isolated from the eyeballs of donors 7–12 (Table S1). A part of the isolated pars plicata was cut by the cross‐section method and cryosectioned. The remaining pars plicata were dissociated and used for fluorescence‐activated cell sorting (FACS) and subsequent experiments. The pars plicata cryosections were subjected to immunofluorescence labeling with the primary antibody mouse monoclonal SOX2 (ab79351, Abcam; 1:200 dilution) at 4°C overnight. Then, the sections were washed carefully and incubated with a Cy3‐labeled goat anti‐mouse IgG (H+L) secondary antibody (Beyotime, A0521) for 1 h. Images were captured by confocal microscopy (LSM 800; Zeiss, Germany).

### 4.5 Fluorescence‐activated cell sorting

The pars plicata was dissociated from the eyeballs of donors 7–12 as previously described and passed through a 30 mm filter. The filtered cell suspensions were resuspended in FACS medium (PBS supplemented with 0.1% BSA), and the number of cells was counted. The cells were transferred into FACS tubes, aliquots of 20,000 cells were removed as unstained and single‐stained controls, and the remainder of the cell suspension was incubated with dialyzed mouse anti‐human TSPAN12‐Alexa Flour 647 (FAB8910R; R&D Systems, dilution 1:20) and mouse anti‐human AQP1‐FITC (orb15121; Biorbyt, dilution 1:50). The cells were incubated on ice in the dark for 30 min. Cells were washed twice with FACS medium (spin at 400 × g for 5 min). Unstained and single‐stained controls were used for proper gating. FACS was then performed on a flow cytometer (S3e; Bio‐Rad, USA). The TSPAN12^+^AQP1^+^ cells were collected in FACS tubes containing 3 ml precooled DMEM.

### 4.6 Stem cell cultures

The stem cell cultures were prepared from donors 7–12 (Table S1). After FACS, the isolated cells were counted and resuspended in a serum‐free medium containing 10 ng/ml fibroblast growth factor 2 (F, F3685; Sigma‒Aldrich), 2 μg/ml heparin (H, B9806; Sigma‒Aldrich) and 20 ng/ml EGF (E, SRP3027, Sigma‒Aldrich). The number of cells was counted after the indicated number of days in culture. To test the clonality of the isolated cells, the cells were plated at a density of one cell per well in individual 96‐well plates in the presence of E+F+H. After 7 days in culture, the wells were inspected for the presence or absence of spheres.

### 4.7 Differentiation and immunostaining

To test the differentiation abilities of the isolated cells from donors 7–12 (Table S1), individual clonally derived spheres were plated in 24‐well plates on a substrate of Matrigel (Corning) in the presence of 10% fetal bovine serum, 10 ng/ml fibroblast growth factor, 2 μg/ml heparin and 20 ng/ml EGF. The medium was changed every 3–4 days. Three weeks later, the plates were fixed with 4% paraformaldehyde for 20 min and permeabilized with 0.3% Triton X‐100 for 10 min. Then, the sections were blocked with goat serum albumin for 60 min at 37°C. After washing with PBS three times, the sections were incubated with primary antibodies at 4°C overnight. Then, the retinas were washed carefully and incubated with secondary antibodies for 1 h. The following primary antibodies were used: mouse monoclonal SOX2 (ab79351; Abcam, 1:200 dilution), rabbit monoclonal Nestin (ab105389; Abcam, 1:200 dilution), mouse monoclonal β‐Tubulin III (ab78078; Abcam, 1:200 dilution), mouse monoclonal Pax2 (H00005076‐M01; NOVUS, 1:500 dilution), rabbit monoclonal GFAP (80788; Cell Signaling Technology, 1:200 dilution), rabbit monoclonal IBA1 (019‐19741; WAKO, 1:100 dilution), rabbit monoclonal rhodopsin (27182; Cell Signaling Technology, 1:200 dilution), mouse monoclonal Nrl (sc‐374277; Santa Cruz Biotechnology, 1:50 dilution), mouse monoclonal RPE65 (sc‐390787; Santa Cruz Biotechnology, 1:50 dilution), mouse monoclonal Calbindin (ab82812; Abcam, 1:200 dilution), mouse monoclonal NF‐M (sc‐32273; Santa Cruz Biotechnology, 1:50 dilution), mouse monoclonal PKC‐alpha (ab32376; Abcam, 1:200 dilution) and mouse monoclonal NeuN (94403; Cell Signaling Technology, 1:500 dilution). The following secondary antibodies were used: Alexa Fluor 488‐labeled goat anti‐rabbit IgG (H+L) (Beyotime, A0423), Alexa Fluor 488‐labeled goat anti‐mouse IgG (H+L) (Beyotime, A0428), Cy3‐labeled goat anti‐mouse IgG (H+L) (Beyotime, A0521), and Cy3‐labeled goat anti‐rabbit IgG (H+L) (Beyotime, A0516).

### 4.8 Statistical analysis

All data are presented as the mean ± SD and were analyzed with SPSS 20.0 software (IBM, Chicago, IL, USA). Data are presented as the means ± SDs. *p*‐Values were calculated using Student's *t*‐test. Differences were considered statistically significant at *p* < 0.05.

## AUTHOR CONTRIBUTIONS

XTW, WF and SPH conceived this study. XTW, WF and ZRX designed and performed the experiments, analyzed the data, and wrote the manuscript. WQL, DL, CYZ, NL and NS helped to conduct in vitro experiments and contributed to data analysis. QZ, GQW, ZZ, SYH and JXH helped to conduct part of the sample collection. XTW, WF, ZRX and RNL analyzed the scRNA‐seq data. SPH conceptualized the study, supervised the experiments and revised the manuscript. All authors read and approved the final manuscript.

## CONFLICT OF INTEREST

The authors declare that they have no conflict of interest.

## ETHICS STATEMENT

The research protocol and written informed consent conformed to the principles of medical ethics. The experimental research program was approved by the Clinical Research Ethics Committee of the First Affiliated Hospital of Chongqing Medical University (approval number. 2019‐099). The study was carried out according to the principles of the Helsinki Declaration. All assays were carried out according to the approved guidelines and regulations.

## Supporting information

Supporting Information

## Data Availability

All data supporting the findings of this study are available from the authors upon reasonable request.
