# Supplementary material for: SOX2‐positive retinal stem cells are identified in adult human pars plicata by single‐cell transcriptomic analyses
Source: MedComm (2020). 2022 Dec 24;4(1):e198. doi: 10.1002/mco2.198 (PMC9790047; doi:10.1002/mco2.198)
Supplement: Supplementary file 1 — Supporting Information [file MCO2-4-e198-s001.docx]

**Supplementary Materials for**

**SOX2-positive retinal stem cells are identified in adult human pars plicata by single-cell transcriptomic analyses**

**Xiaotang Wang^1,2,3,4,^**^#^**, Wei Fan^1,2,3,4,^**^#^**, Zongren Xu^1,2,3,4^, Qi Zhang^1,2,3,4^, Na Li^5^, Ruonan Li^1,2,3,4^, Guoqing Wang^1,2,3,4^, Siyuan He^1,2,3,4^, Wanqian Li^1,2,3,4^, Dan Liao^1,2,3,4^, Zhi Zhang^1,2,3,4^, Nan Shu^1,2,3,4^, Jiaxing Huang^1,2,3,4^, Chenyang Zhao^1,2,3,4^ and Shengping Hou^1,2,3,4,^**^*^

^1^The First Affiliated Hospital of Chongqing Medical University, Chongqing, China

^2^Chongqing Key Laboratory of Ophthalmology, Chongqing, China

^3^Chongqing Eye Institute, Chongqing, China

^4^ Chongqing Branch (Municipality Division) of National Clinical Research Center for Ocular Diseases, Chongqing, China

^5^College of Basic Medicine, Chongqing Medical University, Chongqing 400016, China

^#^These authors contributed equally

*Corresponding author: Shengping Hou Ph.D, The First Affiliated Hospital of Chongqing Medical University, Chongqing 400016, China

Mail address: sphou828@163.com; Telephone number: 0086-23-89011851

**
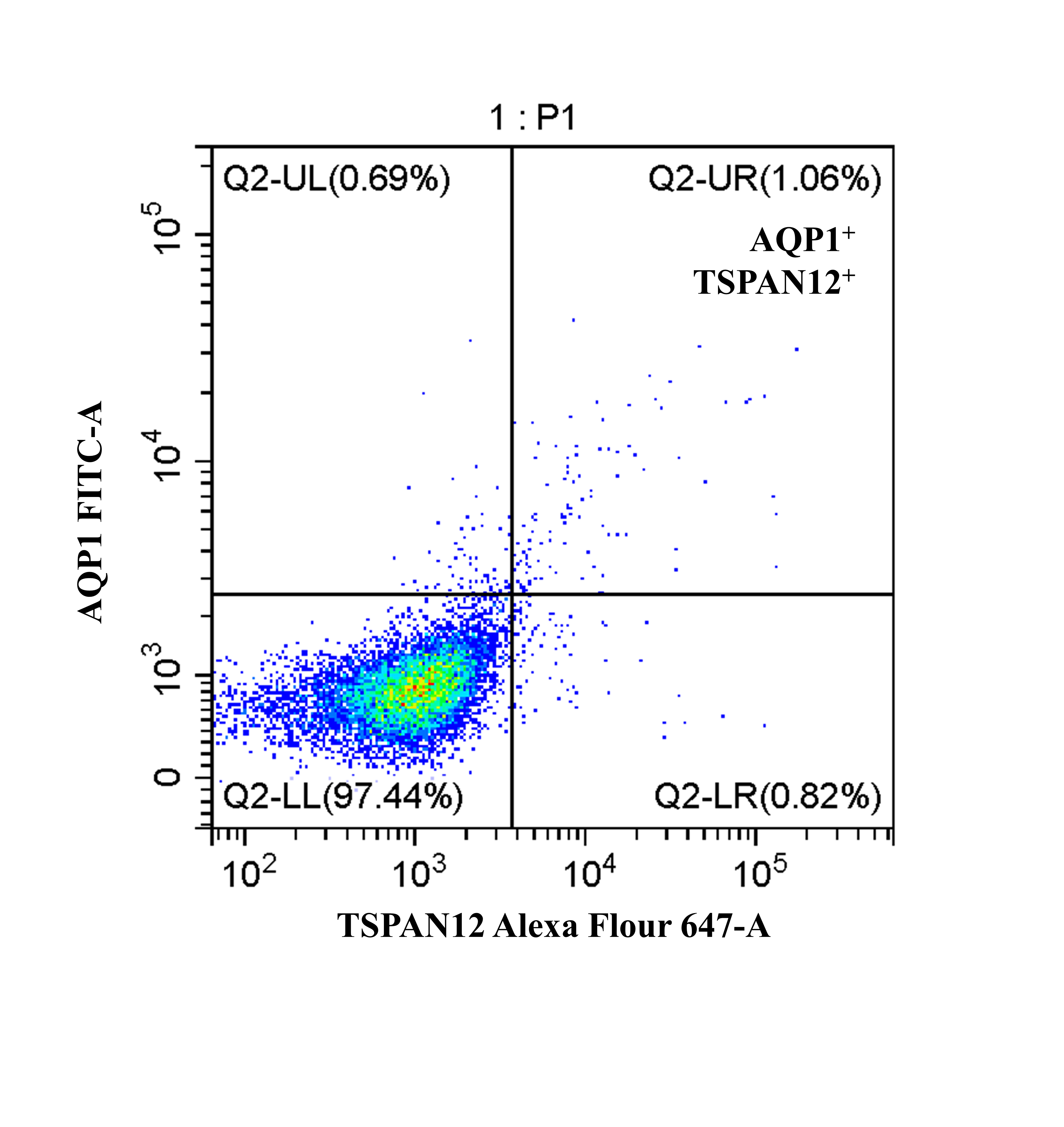
**

**FIGURE S1.** Flowchart of AQP1^+^TSPAN12^+^ cell sorting by FACS.


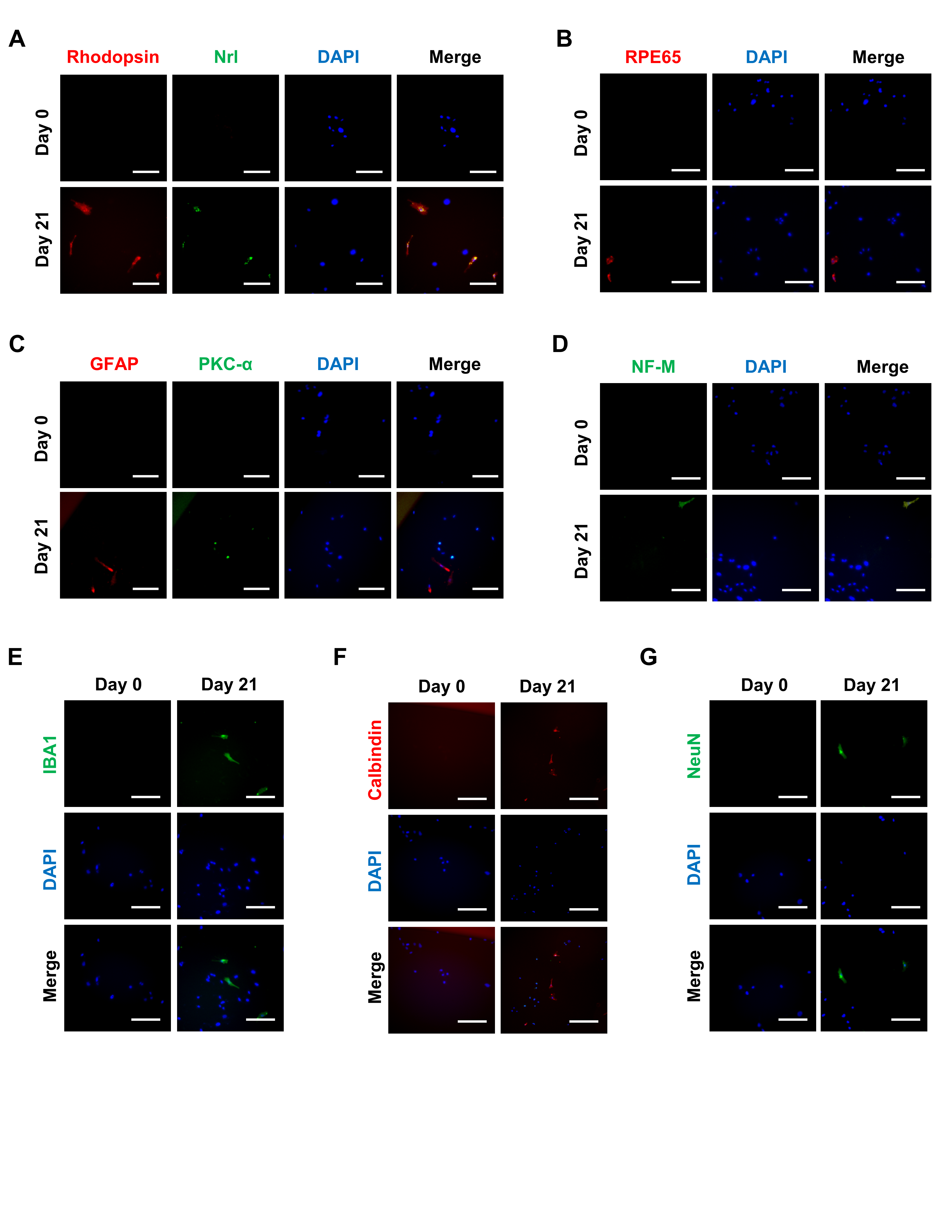


**FIGURE S2.** Representative images of immunofluorescent staining of different cell markers indicate the differentiation abilities of the isolated cells. **A,** Photoreceptor cells (Rho^+^, Nrl^+^). **B,** RPE cells (RPE65^+^). **C,** Astrocytes (GFAP^+^) and bipolar cells (PKC-α^+^). **D,** Ganglion cells (NF-M^+^). **E,** Microglia (IBA1^+^). **F,** Horizontal cells (Calbindin^+^). **G,** Neurons (NeuN^+^). Scale bars: 50 μm (**A-G**). The nuclei of all cells were stained with DAPI.

| **TABLE S1. The information of donors enrolled in the current study** | | | | | |
| --- | --- | --- | --- | --- | --- |
| **Donors** | **Population** | **Post-mortem time** | **Eye disease history** | **Died of** | **Application** |
| Donor 1 | Chinese Han | Within 24 hrs | NO | BD | Single-cell RNA-seq |
| Donor 2 | Chinese Han | Within 32 hrs | NO | HA | Single-cell RNA-seq |
| Donor 3 | Chinese Han | Within 16 hrs | NO | HA | Single-cell RNA-seq |
| Donor 4 | Chinese Han | Within 20 hrs | NO | BD | Single-cell RNA-seq |
| Donor 5 | Chinese Han | Within 24 hrs | NO | BD | Single-cell RNA-seq |
| Donor 6 | Chinese Han | Within 18 hrs | NO | HA | Single-cell RNA-seq |
| Donor 7 | Chinese Han | Within 14 hrs | NO | HA | Validation experiments |
| Donor 8 | Chinese Han | Within 22 hrs | NO | HA | Validation experiments |
| Donor 9 | Chinese Han | Within 8 hrs | NO | BD | Validation experiments |
| Donor 10 | Chinese Han | Within 4 hrs | NO | HA | Validation experiments |
| Donor 11 | Chinese Han | Within 18 hrs | NO | BD | Validation experiments |
| Donor 12 | Chinese Han | Within 24 hrs | NO | HA | Validation experiments |
| BD: Brain Death; HA: Heart Attack | | | | | |
